# Novel Middle-Type Kenyon Cells in the Honeybee Brain Revealed by Area-Preferential Gene Expression Analysis

**DOI:** 10.1371/journal.pone.0071732

**Published:** 2013-08-21

**Authors:** Kumi Kaneko, Tsubomi Ikeda, Mirai Nagai, Sayaka Hori, Chie Umatani, Hiroto Tadano, Atsushi Ugajin, Takayoshi Nakaoka, Rajib Kumar Paul, Tomoko Fujiyuki, Kenichi Shirai, Takekazu Kunieda, Hideaki Takeuchi, Takeo Kubo

**Affiliations:** Department of Biological Sciences, Graduate School of Science, The University of Tokyo, Bunkyo-ku, Tokyo, Japan; Max-Planck-Institut für Neurobiologie, Germany

## Abstract

The mushroom bodies (a higher center) of the honeybee (*Apis mellifera* L) brain were considered to comprise three types of intrinsic neurons, including large- and small-type Kenyon cells that have distinct gene expression profiles. Although previous neural activity mapping using the immediate early gene *kakusei* suggested that small-type Kenyon cells are mainly active in forager brains, the precise Kenyon cell types that are active in the forager brain remain to be elucidated. We searched for novel gene(s) that are expressed in an area-preferential manner in the honeybee brain. By identifying and analyzing expression of a gene that we termed *mKast* (*middle-type Kenyon cell-preferential arrestin-related protein*), we discovered novel ‘middle-type Kenyon cells’ that are sandwiched between large- and small-type Kenyon cells and have a gene expression profile almost complementary to those of large– and small-type Kenyon cells. Expression analysis of *kakusei* revealed that both small-type Kenyon cells and some middle-type Kenyon cells are active in the forager brains, suggesting their possible involvement in information processing during the foraging flight. *mKast* expression began after the differentiation of small- and large-type Kenyon cells during metamorphosis, suggesting that middle-type Kenyon cells differentiate by modifying some characteristics of large– and/or small-type Kenyon cells. Interestingly, *CaMKII* and *mKast*, marker genes for large– and middle-type Kenyon cells, respectively, were preferentially expressed in a distinct set of optic lobe (a visual center) neurons. Our findings suggested that it is not simply the Kenyon cell-preferential gene expression profiles, rather, a ‘clustering’ of neurons with similar gene expression profiles as particular Kenyon cell types that characterize the honeybee mushroom body structure.

## Introduction

The European honeybee (*Apis mellifera* L) is a social insect that forms a sophisticated society. Female adult honeybees differentiate into two castes, reproductive queens and sterile workers [1], [2]. Queens are engaged in laying eggs, while workers shift their labors in an age-dependent manner from nursing their brood (nurse bees) to foraging for nectar and pollen outside the hives (foragers). Foragers transmit information on the distance and direction of a food source to their nestmates using dance communication [1]–[3]. Despite these highly advanced social behaviors, the honeybee brain is rather compact and simple compared to the mammalian brains [4], [5]. The honeybee is therefore an excellent model for studies of the neural and molecular bases of animal social behaviors and higher brain functions [6].

The honeybee brain comprises distinct regions, such as the mushroom bodies (MBs), a higher processing center; the optic lobes (OLs), a visual center; and the antennal lobes (ALs), an olfactory center [4], [7]–[10]. The MBs are a paired structure and each MB has two cup-like structures (calyces). The somata of the intrinsic neurons that comprise the MBs, Kenyon cells (KCs), are located inside (class I KCs) and at the periphery of the calyces [(clawed) class II KCs] [9]. Class I KCs are further classified as large – and small-type KCs based on the size and location of their somata [7], [8]. The somata of large-type KC (lKCs) are located at the inside edges of the calyces, whereas the somata of small-type KC (sKCs) are located in the inner core of the calyces [7].

Genetic studies in *Drosophila* revealed that the MBs are involved in both long – and short-term memory [11]–[14]. In the honeybee, the MBs are involved in associative memory [15]–[17] and higher-order multimodal computations [4], [18]. The proportion of the MBs changes according to the division of labor and/or the foraging experience of the workers [19], [20]. Furthermore, in Aculeata Hymenopteran insects, visual information processed in the OLs projects directly to the MBs, whereas there are few or no direct neural connections from the OLs to the MBs in most other insect taxa [21], [22]. These findings suggest that some brain regions in these insects might have acquired unique functions compared to those of primitive Hymenopteran insects. Although the functional specification of the lKCs and sKCs is not yet clear, we previously identified a novel immediate early gene, that we termed *kakusei*, to show that the central core of the MB calyces, containing mainly sKCs, is active in the forager brains, while the whole KCs are active in the brains of re-oriented bees, suggesting that the sKC-preferential activation is related to information-processing during the foraging flight [23]–[25].

To identify candidate genes related to area-dependent honeybee brain function, we previously used the differential display method, cDNA microarray, matrix-assisted laser desorption ionization-time of flight/mass spectrometry and proteomics to identify genes that are expressed in an area-preferential manner in the honeybee brain [26]–[34]. For example, some genes involved in Ca^2+^-signaling, such as *inositol 1,4,5-trisphosphate receptor* (*IP_3_R*) [26], *Ca^2+^/calmodulin-dependent protein kinase II* (*CaMKII*) [27], *IP_3_ phosphatase* [30], and *reticulocalbin* and *ryanodin receptor* [36] are expressed in an lKC-preferential manner in the honeybee brain, suggesting that Ca^2+^-signaling is enhanced in lKCs. In addition, we previously identified *mblk-1*, which encodes a novel sequence-specific transcription factor that is expressed preferentially in the lKCs [29]. A nematode homolog of the honeybee *mblk-1*, termed *mbr-1*, is also expressed in some sets of neurons and is necessary for pruning excessive neurites during development in *Caenorhabditis elegans* [37], [38].

In contrast, both *ecdysone recepto*r (*EcR*) [33] and its homolog, *HR38* [34], are expressed in an sKC-preferential manner, and the expression of *HR38* is higher in forager brains than in nurse bee brains, suggesting that the ecdysone-signaling in sKCs has a role in the division of labor of workers. Recent genome-wide transcriptomic comparisons of different brain regions in the honeybee revealed that the expression levels of genes involved in signaling and synaptic remodeling are upregulated in the MBs, consistent with our findings that *IP_3_R* and *CaMKII* are more highly expressed in the MBs [39]. Interestingly, in *Drosophila melanogaster*, expression of only few genes among those identified as expressed in a MB-preferential manner in the honeybee brain are enriched in the MBs, suggesting that the MB-preferential expression of the above genes is unique to some Hymenopteran insects, including the honeybee [40].

Among the genes expressed in an MB-preferential manner, *juvenile hormone diol kinase* (*jhdk*), whose product we identified using proteomics, and *tachykinin-related neuropeptide* (*trp*), whose product we identified using matrix-assisted laser desorption ionization–time of flight/mass spectrometry, are unique in that they are preferentially expressed in some lKC subpopulations (we tentatively termed them ‘L1 lKCs’ for *jhdk* and ‘L-a lKCs’ for *trp*, respectively) whose somata are located at the outermost edges of the inside of the MB calyces as well as in sKCs in the worker brain [41], [42]. These findings suggested the existence of other KC subpopulations in the MBs, which could be classified based on their gene expression profiles.

In the latter experiment, however, the area preferentially expressing *mKast* was observed to simply cover the sKCs (Figs. 4F, H), rather than surrounding the upper regions of the sKCs (Fig. 4B, D). The honeybee MB calyces are shaped like baskets whose long sides are rather parallel with the sagittal axis of the brain [49], and the area inside the calyces, where the sKC somata exist, are shaped like cones, whose bases are somewhat elongated along the sagittal axis. Therefore, if frontal brain sections cross the MB calyces at the most front or most backward edges, they sometimes include mainly lKCs [36]. Similarly, it is plausible that, if frontal sections cross the MB calyces in the middle part, mKCs surround the sKCs, as shown in Fig. 4B and 4D, whereas, if the sections cross the MB calyces at the front or backward edges, mKCs merge to simply cover the sKCs, as shown in Figs. 4F and 4H. Actually, in the other case, the area preferentially expressing *mKast* almost occupied inside of the area preferentially expressing *CaMKII* in the lateral calyx (Fig. S2), which corresponded to the far front edge of the lateral MB calyx, whereas it was sandwiched between the area preferentially expressing *CaMKII* and the sKCs in the middle part of the medial MB calyx (Fig. S2, 4B, D).

More recently, to identify genes involved in visual information processing in the honeybee brain, we used a combination of the differential display method and cDNA microarray to search for genes with enriched expression in the OLs of the honeybee brain [35]. Among the genes identified as expressed preferentially in the OLs, we focused on a novel gene, which we termed *middle-type Kenyon cell-preferential arrestin-related protein* (*mKast*). Unexpectedly, expression analysis demonstrated that, in addition to the OLs, *mKast* is expressed preferentially in a restricted area in the MBs, which we termed ‘middle-type KCs’. Our findings revealed a novel honeybee brain area that has never before been recognized in neuroanatomic studies.

## Materials and Methods

### Preparation of bees

European honeybee (*Apis mellifera* L) colonies were purchased from a local dealer (Kumagaya Honeybee Farm, Saitama, Japan) and maintained at The University of Tokyo (Hongo campus). Nurse bees and foragers were collected according to their behaviors, as described previously [43]. Briefly, in-hive worker bees that inserted their heads into larval cells and had well-developed hypopharyngeal glands (glands that synthesize and secrete royal jelly) were collected as nurse bees. Bees returning to the hive entrance with a pollen load on their corbiculae were collected at the hive entrance as forager bees. Queens were purchased from the same local dealer. Worker pupae were collected in the covered comb and their stages were evaluated as described previously [44].

### cDNA microarray analysis

We used a cDNA microarray, which we previously prepared to identify genes expressed in honeybee brain in a brain region – or role-preferential manner [30], to compare gene expression profiles between the OLs and the other brain regions [35]. Briefly, total RNA from the OLs and the other brain regions was divided into 4 groups and two groups were labeled with fluorescent dye Cy5, while the other two groups were labeled with Cy3 (Amersham Bioscience), to prepare two sets of Cy5 – or Cy3-labeled RNA from the OLs and two sets of Cy5 – or Cy3-labeled RNA from the other brain regions. Hybridization was performed twice using a pair of ‘Cy5-labeled OL RNA and Cy3-labeled the other brain region RNA’, and a pair of ‘Cy3-labeled OL RNA and Cy5-labeled the other brain region RNA’. Hybridization was performed twice by exchanging the dyes, Cy5 or Cy3, that were used to label the RNAs, and this hybridization process was repeated to confirm the results.

We calculated the ratio of the expression level of each clone in the OLs relative to that in the remaining brain regions and looked for clones whose ratios were greater than 1.4-fold. 45 independent clones were identified as candidate genes whose expression was more enriched in the OLs than in the other brain regions. Expression analysis of 19 clones selected arbitrarily from the 45 identified candidate clones performed using *in situ* hybridization with a DIG-labeled RNA probe led to the identification of three clones, two of which we previously characterized as *Clones #1* and *#2* (GenBank accession Nos. BP538943 and BP539264), that were strongly expressed in the OLs compared with the other brain regions [35]. In the present study, we characterized the remaining *Clone #3* (GenBank accession no. BP874957.1).

### Phylogenetic tree analysis

Genomic sequences of the honeybee, *Drosophila*, *C. elegans*, and human origin, whose predicted products showed significant sequence similarities (approximately more then 25%) with the arrestin-like_C domain of mKast, were obtained by an NCBI database search. In addition, genomic sequences for known or predicted arrestins of the honeybee, *Drosophila*, and human origin were also obtained by an NCBI database search. A non-rooted phylogenetic tree was constructed with the nucleotide sequences of the arrestin-like_C domains of the above-mentioned genes using the neighbor-joining method [45] and ‘Clustal X’ software.

### *In situ* hybridization analysis

For preparation of probes, total RNA was extracted from the brain samples using TRIzol Reagent (Invitrogen), treated with DNase I (Invitrogen), and then reverse-transcribed with SuperScript III (Invitrogen) using an oligo (dT) primer. RT-PCR was performed using the following gene specific primer pairs: M13 forward and reverse primers for *Clone #3*/*GB18367* (*mKast*); gtggcgcgagaattctacag and ccacagtctcttgtctgtgg for *CaMKII*; gaatttcaaatttcgcctcgacg and ttttggaacaaccccaccatc for *Mblk-1*; tcgatttcaatcagagtggcg and cggctcgtccgaggagaaatattg for *jhdk*; gcccaatcgacgacgttatc and cccctcgttccataaaatccc as forward and reverse primers for *Trp*, respectively. PCR products were then subcloned with a pGEM-T Easy Vector System (Promega), and sequenced using a Big Dye Terminator v3.1 Cycle Sequencing Kit (Applied Biosystems). Plasmids containing the fragment cDNA for *Clone #3* (GenBank accession nos. BP874957, corresponds to the 3'UTR of *GB18367*), *CaMKII*, *mblk-1*, *jhdk*, and *trp* were re-amplified by PCR with M13 forward and reverse primers, respectively. The PCR products containing the T7 and SP6 promoter sites were purified using a PCR Purification Kit (Qiagen). The DIG-labeled or biotin-labeled sense and antisense RNA probes were prepared by *in vitro* transcription using a DIG RNA labeling kit and a biotin RNA labeling kit (Roche).

For *in situ* hybridization, the bees were anesthetized on ice and their heads were removed with fine scissors. The whole brains were dissected from the heads and mounted in Tissue-Tek OCT compound (Sakura Finetechnical Co) on dry ice. Frozen vertical brain sections (10 µm thick) were prepared using a microtome, and placed on slides coated with 3-aminopropyl-triethoxysilane (Matsunami), air-dried at room temperature overnight, and subjected to *in situ* hybridization.

The sections on slides were fixed in 4% paraformaldehyde in 100 mM sodium phosphate buffer (PB) at pH 7.4 at 4°C overnight, treated with 10 µg/ml proteinase K in TE buffer (10 mM Tris-HCl buffer, pH 8.0, containing 1 mM EDTA) for 15 min at room temperature, re-fixed in 4% paraformaldehyde in PB for 15 min at 4°C, and then treated in 0.2 M HCl for 10 min. The sections were placed in 0.1 M triethanolamine-HCl buffer, pH 8.0, containing 0.1 M acetic acid anhydride for 10 min, and then washed with PB for 1 min at room temperature. After dehydration through a graded series of ethanol (70%, 80%, 90%, and 100%), brain or head sections were hybridized overnight with DIG-labeled riboprobes (1∶50 dilution) and/or biotin-labeled riboprobes (1∶2500 dilution) at 60°C.

DIG-labeled riboprobes were diluted with hybridization buffer (10 mM Tris-HCl buffer, pH7.6 containing 50% formamide, 200 mg/ml tRNA, and 1x Denhardt's solution [0.02% Ficoll, 0.02% polyvinylpyrrolidone, and 0.02% bovine serum albumin], 10% dextran sulfate, 600 mM NaCl, 0.25% sodium dodecyl sulfate, and 1 mM EDTA) and preincubated for 10 min at 85°C. After hybridization at 60°C overnight in a moist chamber, the sections were washed with 5x saline sodium citrate (SSC) buffer and 2x SSC containing 50% formamide at 60°C for 30 min, treated with 10 mg/ml RNase A (Sigma) in TNE (10 mM Tris-HCl pH7.5; 1 mM EDTA; 0.5 M NaCl) buffer by incubating for 30 min at 37°C, followed by washing with TNE buffer at 37°C for 10 min, and successively washed with 2x SSC and 0.2 x SSC twice for 20 min at 60°C.

DIG-labeled riboprobes were detected immunocytochemically with alkaline phosphatase-conjugated anti-DIG antibody using a HNPP Fluorescent Detection Set (Roche), and biotin-labeled riboprobes were detected with TSA plus System (Perkin Elmer) according to the manufacturer's instructions. As a negative control, sections were hybridized with sense probes and the antisense probe-specific signals were confirmed in every experiment. Micrographs of fluorescent *in situ* hybridization were taken using a fluorescent microscope (Axio Imager Z1, Carl Zeiss). 4′,6-Diamino-2-phenylindole, dihydrochloride (DAPI, Invitrogen) was used to stain the nuclear DNA. Intensity and brightness of the micrographs were processed using Photoshop CS software (Adobe Systems).

### *In situ* hybridization of *kakusei*

*In situ* hybridization of *kakusei* was performed as described previously [23]–[25]. Frozen coronal brain sections (10 µm thick) were fixed in 4% paraformaldehyde in phosphate buffered saline, pretreated, and hybridized with DIG-labeled riboprobes. The DIG-labeled riboprobes were synthesized by T7 or SP6 polymerase with a DIG labeling mix (Roche) from a template containing the fragment isolated by differential display (from +4511 to+5159) [23]. After stringent washes, DIG-labeled riboprobes were detected immunocytochemically with peroxidase-conjugated anti-DIG antibody (1∶500; Roche) and TSA Biotin System (Perkin Elmer). Sense probes were used as negative controls and the signals were confirmed to be antisense probe-specific in every experiment. Micrographs of fluorescent *in situ* hybridization were taken using an IX71 confocal microscope (Olympus). Intensity and brightness of the micrographs were processed with Photoshop software (Adobe).

### Quantitative reverse transcription polymerase chain reaction (RT-PCR) of *mKast*

Quantitative RT-PCR was performed essentially as described previously [46]. After the OLs and the other brain regions (for Fig. S1), and brain regions that mainly contained the MBs (for Fig. S4) were dissected from the nurse bees and foragers, they were homogenized with a bead cell crusher (MS-100; Tomy, Tokyo, Japan). Total RNA extracted using TRIzol reagent was reverse transcribed with a PrimeScript RT reagent Kit (Takara) and quantitative RT-PCR was performed with LightCycler (Roche, Nutley, NJ) using SYBR Premix Ex TaqII (Takara) and gene-specific primers for *mKast*; tccagcagtaccgttgtacg and cgagtacggcttgacctctc, *EF-1alpha*; ttggtttaagggatggactg and ccatacctggtttcaacaca. PCR products of *mKast* and *ef-1α* of known concentrations were used as standards. The amount of *mKast* transcript was normalized with that of *EF-1alpha*. Tukey-Kramer's test was performed to examine the significant difference in the relative expression of *mKast* among nurse bees and foragers using JMP software (SAS, Cary, NC). There was no significant difference in the expression of *EF-1alpha* between the brains of nurse bees and foragers (data not shown).

## Results

### Identification of *mKast*, which is expressed preferentially in the OLs and a part of the MBs in the honeybee brain

**Figure 1:**
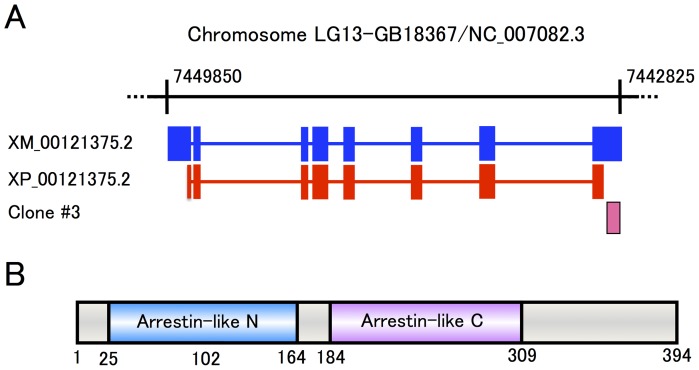
Gene structure and primary structure of *mKast* (GB18367/NC_007082.3). Gene structure of *mKast* (GB18367/NC_007082.3). The exon (blue closed boxes)/intron (blue horizontal lines) structure of *mKast* is presented below the corresponding part of Chromosome LG13. Note that *mKast* is located in the reverse strand. Numbers indicate the positions of the start and end nucleotides of *mKast* in Chromosome LG13. XM_00121375.2 indicates the mRNA structure of *mKast*, which corresponds to all of the exons (blue boxes), whereas XP_00121375.2 indicates the coding regions of *mKast*, which correspond to parts of the first and eighth exons and all of the second to seventh exons (red boxes). Clone #3 (magenta box) corresponds to almost the entire 3–UTR of the eighth exon. (B) Domain structure of mKast. Arrestin-like_N and _C domains are indicated in the primary structure (gray box) of mKast. Numbers indicate amino acid positions.

We previously used a combination of the differential display method and cDNA microarray to identify *Futsch* and *MESK2* whose expression in enriched in the OLs in the honeybee brain [35]. We continued our efforts to identify a gene fragment *Clone #3* (GenBank accession no. BP874957.1; 754bp), whose expression in the OLs was approximately 1.8 fold higher than in the other brain regions, that corresponds to almost the entire 3′-UTR of the eighth exon of GB18367/NC_007082.3 (Fig. 1A). Subsequent quantitative RT-PCR analysis revealed that expression of *Clone #3* in the OLs was approximately 2.5 fold higher than in other brain regions (Fig. S1), which was consistent with the results of cDNA microarray analysis. GB18367 is an uncharacterized gene located in Chromosome LG13 that encodes a predicted protein termed ‘arrestin domain-containing protein 2 (tentatively abbreviated Ardp2)’ or ‘*Apis mellifera* arrestin-like 4 (ArrL4)’, comprising 394 amino acid residues (Fig. 1).

**Figure 2:**
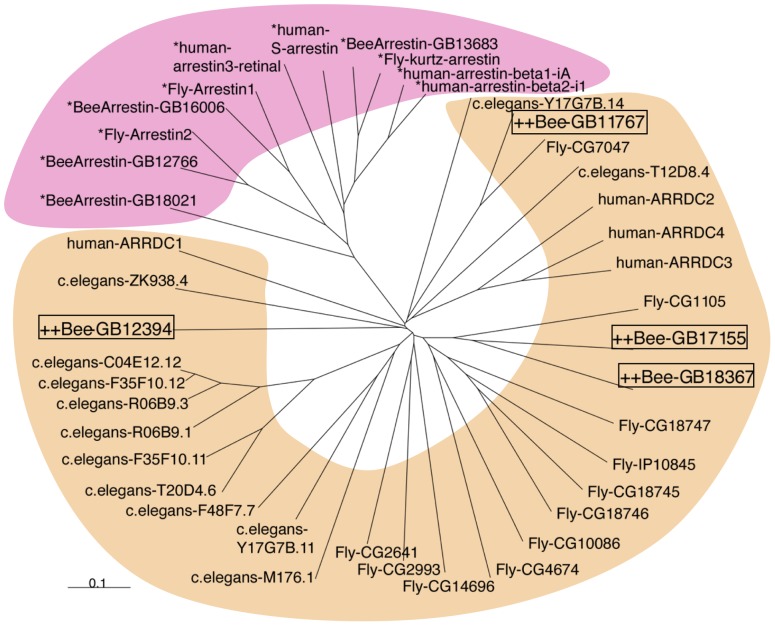
Phylogenetic tree analysis of genes containing arrestin-like domains. A phylogenetic tree was constructed using the neighbor-joining method [51]. Known arrestin genes and other genes that encode proteins with arrestin-like domains of *Apis mellifera* (Bee-) *Drosophila melanogaster* (Fly-), *Caenorhabditis elegans*, (c. elegans-)and *Homo sapiens* (human-) origin are indicated by the pink and orange background color, respectively. The scale bar corresponds to 0.1 substitutions/site.

Although the GB18367 product contained both arrestin-like_N and arrestin-like_C domains (Fig. 1B), which are contained in arrestins [47], neither of these domains had any significant sequence identities with those of *Apis mellifera* arrestin homolog isoform 1 (GB16006), isoform 2 (GB12766), or β-arrestin (GB13683), three arrestin homologs predicted in the honeybee genome, suggesting that the GB18367 product is structurally not closely related to arrestins. Rather, the GB18367 product had low but significant sequence identities with mammalian arrestin domain-containing proteins (ARRDCs) 1–4: e.g., 26% and 27% with human and mouse ARRDC2s, respectively (Fig. 2), whose functions have not been well characterized [48].

In addition to GB18367, there are three significantly related and uncharacterized genes in the honeybee genome: GB17155 (predicted to encode Ardp3), GB12349 (also predicted to encode Ardp3), and GB11767 (predicted to encode Ardp2), whose products have 38%, 28% and 27% sequence identities with the GB18367 product, respectively. All of these genes are predicted to encode proteins with both arrestin-like N and arrestin-like C domains. In contrast, there are some Hymenopteran genes more closely related to GB18367: XP 003694543.1 in *Apis florea*, XP 003494477.1 in the bumblebee *Bombus terrestris*, XP 003703224.1 in the alfalfa leafcutter bee *Megachile rotundata* and XP 001605392.2 in *Nasonia vitripennis*, whose products have 97%, 85%, 82% and 56% sequence identities with the GB18367 product. Genes that encode proteins with lower sequence identity (less than 33%) with the GB18367 are present in some other insect species, including the yellow fever mosquito *Aedes aegypti* (Aael_AAEL008185; 33% sequence identity) and the fruit fly *Drosophila melanogaster* (CG1105; 28% sequence identity), as well as other invertebrates, including the Giant Pathyfic oyster *Crassostrea gigas* (EKC20632.1; 32% sequence identity) and *Caenorhabditis elegans* (NP_499816.2; 28%). These findings suggested that, while there are three less related homologs in the honeybee genome, GB18367 represents a gene that is unique to Aculeata Hymenopteran insects. On the other hand, phylogenic tree analysis revealed that GB18367 belongs to a protein superfamily that is distinct from the arrestin family, conserved beyond animal species and comprises honeybee GB17155, GB12349 and GB11767 as well as *Drosophila* CG1105 and mammalian ARRDC genes (Fig. 2).

Because of the confusing nomenclature for these proteins: i.e., two different names (Ardp2 and ArrL4) were given to the same gene product (GB18367), the same name (Ardp2) was given to two different gene products (GB18367 and GB11767), GB18367 and its homologs seemed to be unique to some Hymenopteran insects, and there is no apparent GB18367 ortholog in mammalian ARRDCs, we renamed GB18367 ‘*mKast*’ based on the results of our gene expression analyses.

**Figure 3:**
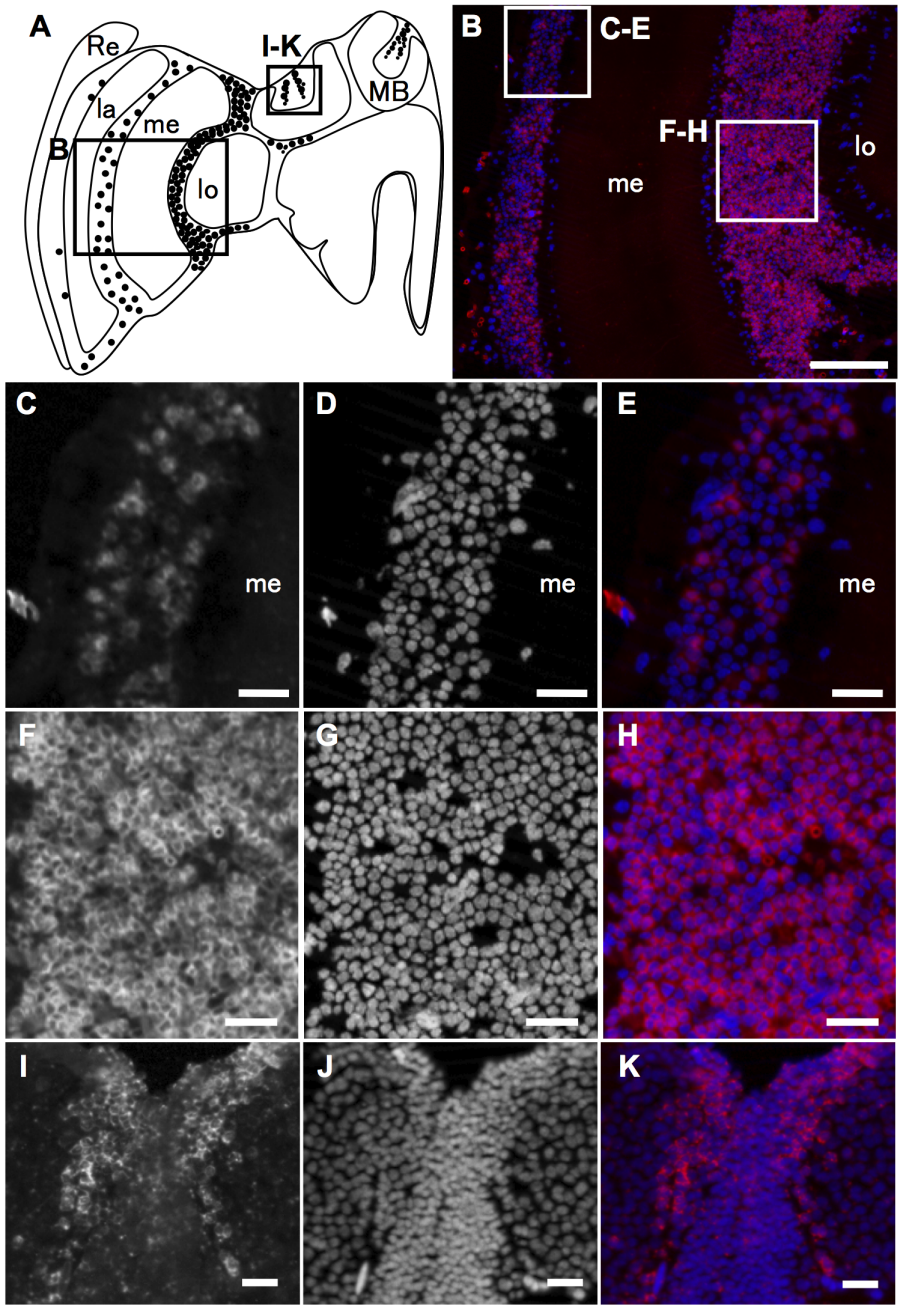
Single fluorescent *in situ* hybridization of *mKast* in the worker brain. (**A**) Schematic drawing of the distribution of the *mKast*-expressing cells in a sagittal left hemisphere section of a worker brain. *mKast*-expressing cells (black dots) were scattered between the lamina (la) and medulla (me), whereas almost all of the cells located between the medulla (me) and lobula (lo) expressed *mKast*. (**B**) Results of single *in situ* hybridization with *mKast* antisense probe in a worker brain area corresponding to box (B) shown in panel (A). *mKast* expression is indicated in magenta, whereas nuclei were stained with DAPI (blue). (**C–H**) Magnified views of boxes (C–E) and (F–H) in panel (B). (**I–K**) Magnified views of box (I–K) in panel (A). Signals of *mKast* (C, F, I) and DAPI (D, G, J), and merged images of signals of *mKast* and DAPI are shown in independent panels, respectively. la, lamina; lo, lobula; me, medulla; OL, optic lobe; MB, mushroom body; Re, retina. Bars represent 100 µm in panel (B) and 20 µm in panels (C–H), respectively.

Although we originally identified *mKast* as a gene, whose expression is more enriched in the OLs than in the other brain regions, subsequent analyses suggested that *mKast* was also expressed preferentially in a restricted region of the MBs. Although this did not support our aim to identify an OL-preferential gene, *mKast*-expression in the MBs appeared unique, raising the possibility that analysis of its expression in the MBs could lead to the discovery of a novel MB structure. Thus, we used single fluorescent *in situ* hybridization to more closely analyze the cell types that preferentially express *mKast* in both the OLs and MBs. In the OLs, cells preferentially expressing *mKast* were scattered throughout the lamina-medulla layers (Figs. 3A–E), whereas most of the neurons in the medulla-lobula layers preferentially expressed *mKast* (Fig. 3A, B, F–H). In the MBs, the expression pattern of *mKast* was quite unique: it was preferentially expressed in two vertical stripes inside each MB calyx (Fig. 3A, I–K). Preferential expression of *mKast* was observed at the interface of the lKCs and sKCs, whose somata are located at the edges and the inner core of the MB calyces, respectively.

### Comparison of the *mKast*-expression profile with those of *CaMKII* and *Mblk-1*, which are expressed in an lKC-preferential manner

We then hypothesized that the area preferentially expressing *mKast* could be sandwiched by lKCs and sKCs and correspond to an area, in which expression of *trp* and *jhdk* is not enriched. Therefore, we used fluorescent double *in situ* hybridization with worker brain sections to compare KCs preferentially expressing *mKast* and those preferentially expressing *CaMKII* or *mblk-1* [27], [29], and those expressing *trp* or *jhdk*, which are preferentially expressed in both lKCs and sKCs, but not in lKCs at the inner edges (‘L-2’ and ‘L-b’ lKCs, respectively) [41], [42].

**Figure 4:**
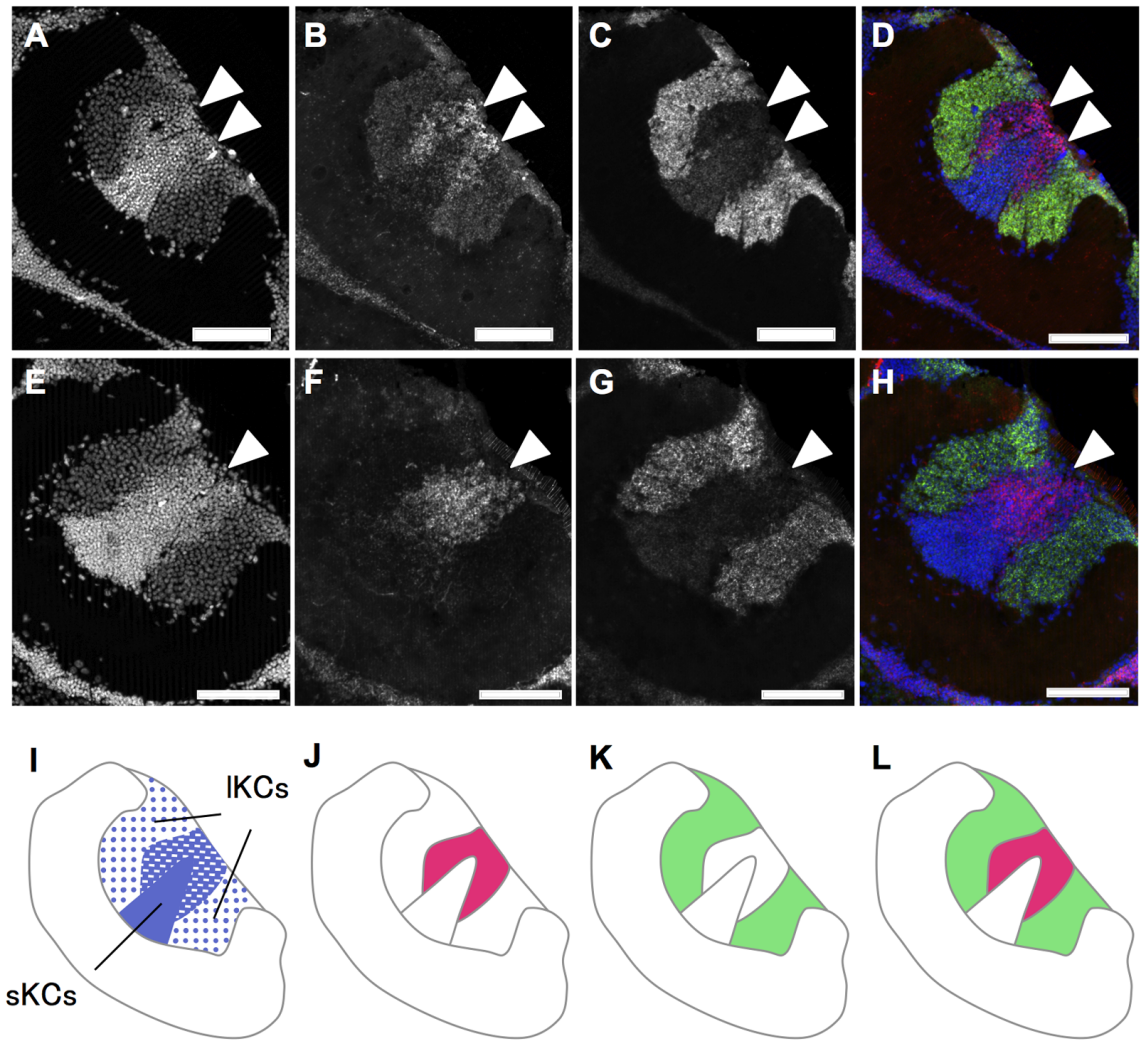
Double fluorescent *in situ* hybridization of *mKast* and *CaMKII* or *mblk1* in the worker brain. (**A–D**) Double *in situ* hybridization with *CaMKII* and *mKast* antisense probes. Nuclear signals detected by DAPI (A), *mKast* signals detected by HNPP/FastRed (B), *CaMKII-*signals detected by fluorescein (C) and merged images of the two (D) are shown. In the merged image (D), DAPI signals, *mKast* signals and *CaMKII* signals are colored in blue, magenta, and green, respectively. White arrowheads indicate regions with *mKast* signals. Bars indicate 100 µm. (**E–H**) Double *in situ* hybridization with *mblk-1*and *mKast* antisense probes. Nuclear signals detected by DAPI (E), *mKast* signals detected by HNPP/FastRed (F), *mblk-1* signals detected by fluorescein (G) and merged images of the two (H) are shown. In the merged image (H), DAPI signals, *mKast* signals and *mblk-1* signals are colored blue, magenta, and green, respectively. White arrowheads indicate regions with *mKast-*signals. (**I–L**) Schematic drawings of the signal pattern corresponding to the above two panels, respectively. Signals of DAPI, *mKast*, *CaMKII* or *mblk-1* are colored blue, magenta, and green, respectively. lKCs, large-type Kenyon cells; sKCs, small-type Kenyon cells.

We first performed double fluorescent *in situ* hybridization of *mKast* and *CaMKII*. Preferential *mKast* expression was observed just adjacent to the lKCs preferentially expressing *CaMKII*, and there was no significant overlap between the areas preferentially expressing *mKast* and *CaMKII*, respectively (Fig. 4A–D, I–L), indicating that lKCs do not preferentially express *mKast*. We then examined whether the area preferentially expressing *mKast* overlapped with that of *Mblk-1*. Preferential *mKast* expression was again observed just adjacent to the lKCs preferentially expressing *Mblk-1*, and there was again no significant overlap between the areas preferentially expressing *mKast* and *Mblk-1*, respectively (Fig. 4E–H), confirming that lKCs do not preferentially express *mKast*. In both experiments, there was also no overlap between the area preferentially expressing *mKast* and the sKCs, whose somata were densely stained at the innercore of the MB calyces (Fig. 4A–L).

Considerable *mKast* expression was also observed in some neurons, whose somata are located at the periphery of the calyces, in some specimens (e.g., Fig. 4F, H, bottom right corner), possibly reflecting individual differences in *mKast* expression in these neurons.

### Comparison of *mKast*-expression profile with *jhdk* and *trp*, which are preferentially expressed in both lKCs and sKCs

**Figure 5:**
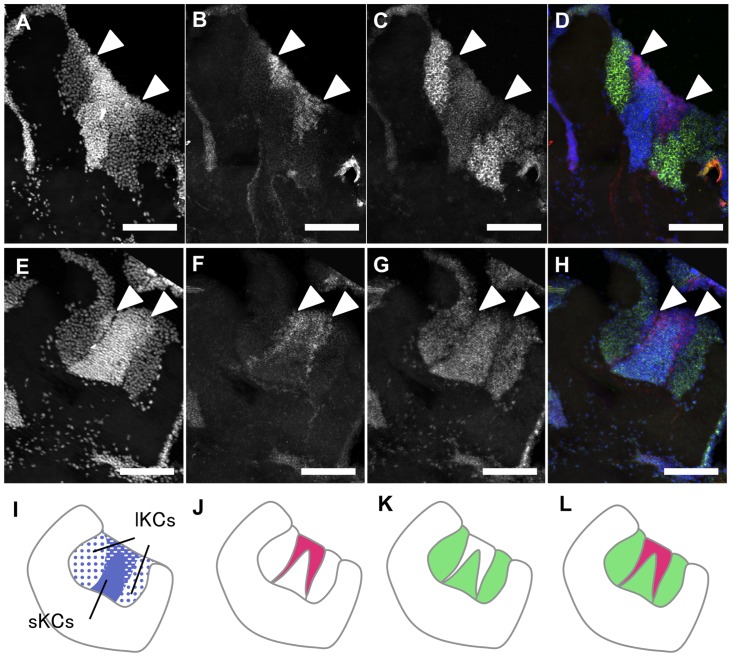
Double fluorescent *in situ* hybridization of *mKast* and *jhdk* or *trp* in the worker brain. (**A–D**) Double *in situ* hybridization with *jhdk* and *mKast* antisense probes. Nuclear signals detected by DAPI (A), *mKast* signals detected by HNPP/FastRed (B), *jhdk-*signals detected by fluorescein (C) and merged images of the three (D) are shown. In the merged image (D), DAPI signals, *mKast* signals and *jhdk* signals are colored blue, magenta and green, respectively. White arrowheads indicate regions with *mKast* signals. Bars indicate 100 µm. (**E–H**) Double *in situ* hybridization with *trp* and *mKast* antisense probes. Nuclear signals detected by DAPI (E), *mKast* signals detected by HNPP/FastRed (F), *trp* signals detected by fluorescein (G) and merged images of the two (H) are shown. In the merged image (H), DAPI signals, *mKast* signals and *trp* signals were colored blue, magenta, and green, respectively. White arrowheads indicate regions with *mKast-*signals. (**I–L**) Schematic drawings of the signal pattern corresponding to the above two panels, respectively. Signals of DAPI, *mKast*, and *jhdk* or *trp* are colored blue, magenta and green, respectively. lKCs, large-type Kenyon cells; sKCs, small-type Kenyon cells.

We next performed fluorescent double *in situ* hybridization analysis for *mKast* and *jhdk* or *trp* using worker brain sections. The areas preferentially expressing *mKast* and *jhdk*, respectively, did not overlap, indicating that KCs preferentially expressing *mKast* are not sKCs and correspond to ‘L-b’ lKCs, in which *jhdk* expression is not enriched (Fig. 5A–D, I–L). Similarly, the areas preferentially expressing *mKast* and *trp*, respectively, did not overlap, indicating that KCs preferentially expressing *mKast* are not sKCs and correspond to ‘L-2’ lKCs, in which *trp* expression is not enriched (Fig. 5E–H). Note that, in the latter experiment, the area preferentially expressing *mKast* was again detected to mostly cover the sKCs (Fig. 5F, H). These findings clearly indicated that the area preferentially expressing *mKast*, which corresponds to the previous ‘L-2’/‘L-b’ lKCs, represents a novel MB area, in which none of the above four genes so far identified as being expressed in an lKC – or sKC-preferential manner are preferentially expressed.

**Figure 6:**
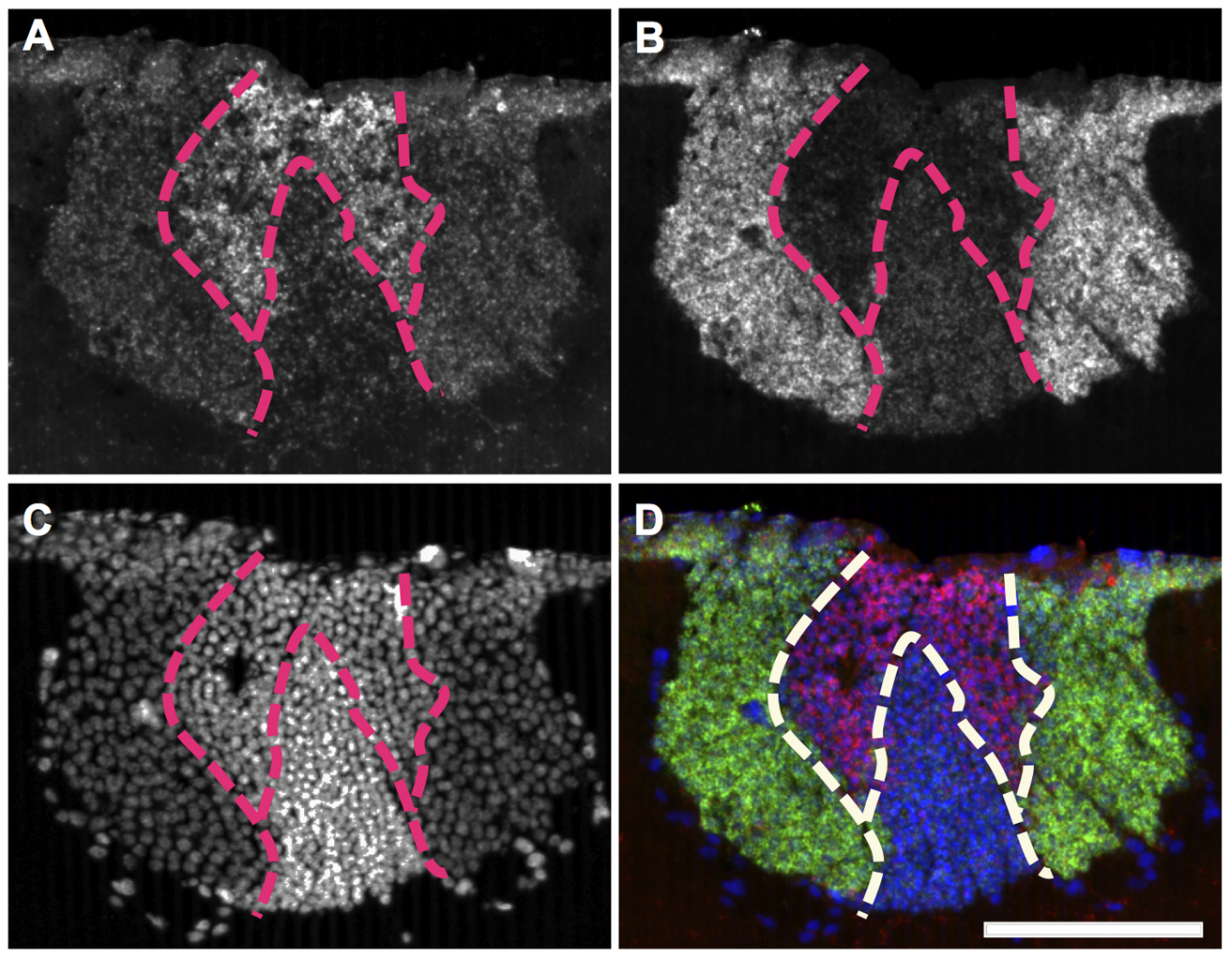
Identification of mKCs that are characterized by *mKast* expression. (**A–D**) The same double *in situ* hybridization result with *CaMKII* and *mKast* antisense probes shown in Fig. 3 (D). (**A**) *mKast-*signals detected by HNPP/FastRed, (**B**) *mblk-1-*signals detected by fluorescein, (**C**) nuclear signals detected with DAPI, and (**D**) merged images of the three. Dashed lines indicate borders between cells expressing *mKast* mKCs, magenta) and cells expressing *mblk-1* (lKCs, green), and cells expressing *mKast* (mKCs) and cells stained with DAPI (sKCs, blue). The bar indicates 100 µm.

We termed the KCs contained in this area as the ‘middle-type KCs (mKCs)’, because the area is sandwiched just between the lKCs, which preferentially express *CaMKII* (Fig. 6A, B, D), and sKCs, whose somata are localized at the inner core of the calyces (Fig. 5A, C, D), and because the size of the ‘mKC’ somata is just intermediate between that of sKCs (mean 5–7 µm) and lKCs (mean 7–9 µm) [8] (Fig. 6A–D). Similar expression profiles of *mKast* were observed by single fluorescent *in situ* hybridization using brain sections of nurse bees and foragers (Fig. S3), and the *mKast* expression level determined by quantitative RT-PCR did not differ significantly among the brains of nurse bees and foragers (Fig. S4), suggesting that this novel brain area functions are not associated with the division of labor of workers.

### Expression analysis of *mKast* in the OLs of the honeybee brain

**Figure 7:**
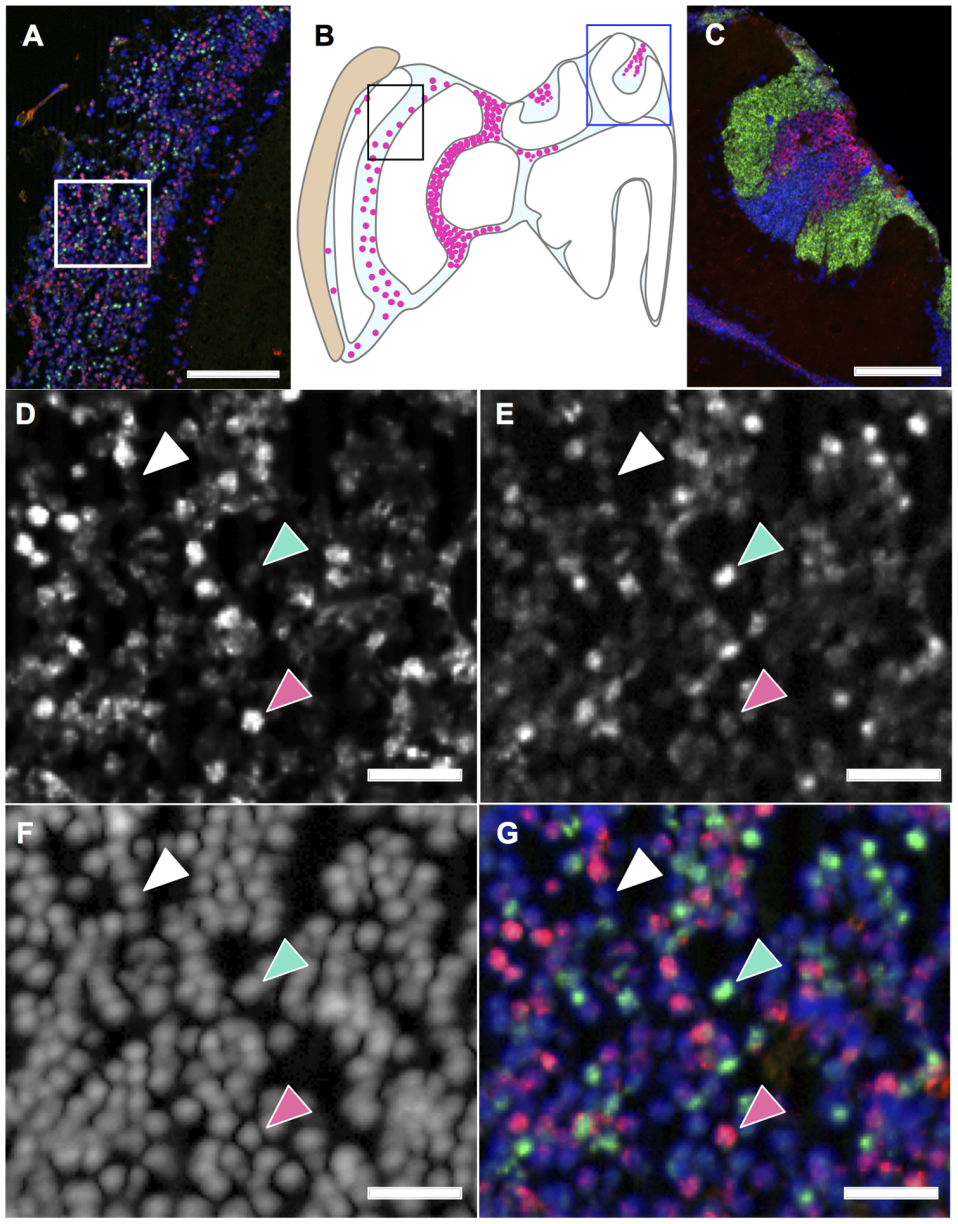
Expression analysis of *mKast* and *CaMKII* revealed a novel ‘module-like structure’ in the honeybee brain. (**A**) Double *in situ* hybridization of *CaMKII* and *mKast* in the worker OL area. Nuclear signals detected by DAPI, *mKast* signals detected by HNPP/FastRed and *CaMKII*-signals detected by fluorescein are colored blue, magenta, and green, respectively. (**B**) Schematic drawing of the distribution of *mKast*-expressing cells (red dots) in a left hemisphere section of the worker brain. (**C**) Double *in situ* hybridization of *CaMKII* and *mKast* in the worker MB area. Nuclear signals detected by DAPI, *mKast* signals detected by HNPP/FastRed, and *CaMKII* signals detected by fluorescein are colored blue, magenta and green, respectively. (**D–E**) Magnified views of the box shown in panel (A). (**D**) Nuclear signals detected by DAPI, (**E**) *mKast* signals detected by HNPP/FastRed, (**F**) *CaMKII* signals detected by fluorescein, and (**G**) a merged image of three. Note that there are three types of cells expressing either *mKast* (indicated by red arrowheads), *CaMKII* (indicated by green arrowheads) or neither (indicated by white arrowheads) in panels (D–G). Bars indicate 100 µm in panels (A and C) and 20 µm in panels (D–G).

We originally identified *mKast* as a gene whose expression is enriched in the OLs. Therefore, we questioned whether expression of *CaMKII* is also enriched in a restricted set of OL neurons, and in that case, is there any overlap between the OL neurons preferentially expressing *mKast* and *CaMKII*, respectively. For this, we evaluated the OL regions of the same *in situ* hybridization specimens that we used to examine the expression of *mKast* and *CaMKII* in the MBs (Fig. 4). We found that the neurons preferentially expressing *CaMKII* were also scattered throughout the OLs, although the number of neurons preferentially expressing *CaMKII* was much lower than that of neurons preferentially expressing *mKast* (Fig. 7A, B). Interestingly, neurons preferentially expressing *mKast* and *CaMKII*, respectively, in the OLs did not overlap, and each gene was preferentially expressed in distinct sets of OL neurons (Fig. 7A, E–G). In addition, many OL neurons expressed neither *mKast* nor *CaMKII* preferentially and were stained with DAPI, similar to sKCs (Fig. 7A, D–G). These results suggested that it is not simply the KC-preferential gene expression profiles, rather, a ‘clustering’ of neurons with similar gene expression profiles as particular KC types that characterize the honeybee mushroom body structure.

### sKCs and some mKCs are active in forager brains

**Figure 8:**
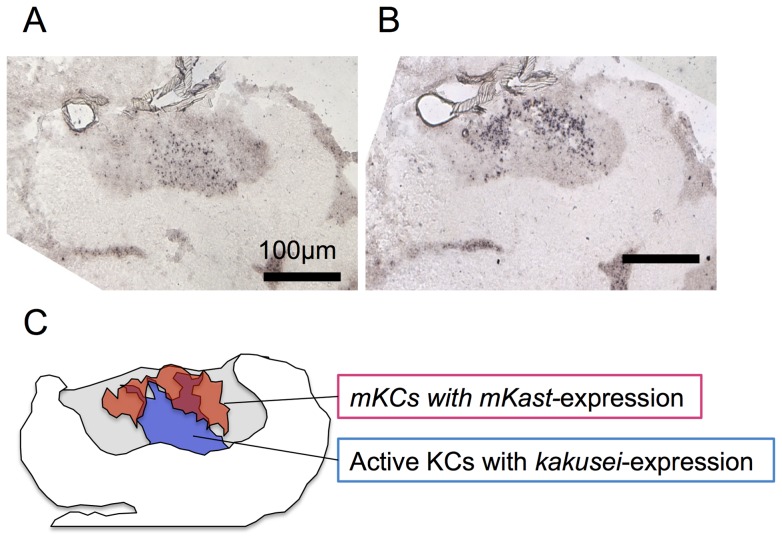
Activity mapping of mKCs and sKCs in the forager brain. (A) *In situ* hybridization of *kakusei*, an immediate early gene, to map active KCs in the forager brain. Note that *kakusei* signals (purple dots) were detected in the inner region of the MB calyx. (B) *In situ* hybridization of *mKast* to identify mKCs in the forager brain. Note that *mKast*-signals (purple dots) represent the localization of mKCs. (C) Schematic drawing of the distribution area of mKCs expressing *mKast* (orange) and active KCs expressing *kakusei* (blue). Note that not only sKCs (blue area) but also some mKCs (merged red area) are active in the forager brain. Bars indicate 100 µm.

We next examined the potential role(s) of mKCs in honeybee social behavior. We previously used a novel immediate early gene termed *kakusei* to map active brain regions in the forager brains [23]–[25]. Although we previously reported that the neural activity of ‘sKCs is enhanced in the forager brain’ [23], the upper and lower edges of the MB area expressing *kakusei* actually stretch, whereas the somata of sKCs are located at the inner core of the calyces just like a cone [33], [34]. Therefore, we thought that the active MB area in the forager brain might correspond to sKCs and some mKCs. To test this hypothesis, we compared the area preferentially expressing *kakusei* and *mKast* in the MBs by single *in situ* hybridization analysis with serial worker brain sections. Fluorescent double *in situ* hybridization could not be applied in this experiment because the level of *kakusei* expression was much lower than that of *mKast*, and thus it was difficult to show their expression at a similar level in the same section. The *kakusei*-expressing area corresponded to the entire area of sKCs and the inner area of mKCs (Fig. 8), supporting our notion that the neural activity of both sKCs and some mKCs is enhanced in the forager brains.

### mKCs differentiate after lKC and sKCs proliferation ceases during metamorphosis

**Figure 9:**
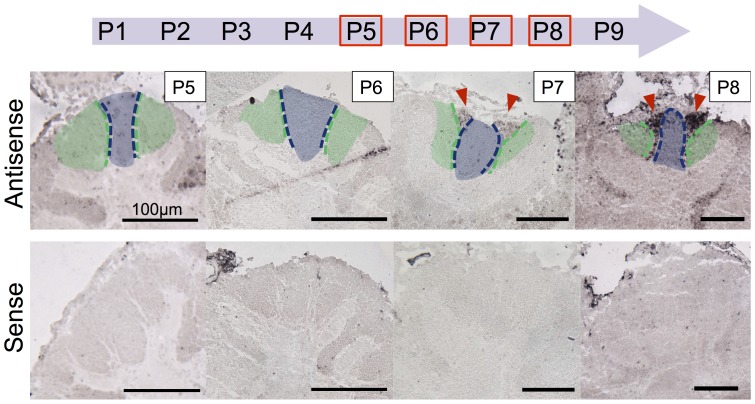
*mKast* expression begins after the proliferation of lKCs and sKCs ceases. (Upper arrow) Staging of the honeybee metamorphosis. P1–P9 in the arrow colored in light purple indicate the pupal stages. P5–P8 stages analyzed by in situ hybridization of *mKast* are boxed with red lines. (Middle panels labeled with P5–P6) *In situ* hybridization with *mKast*-antisense probe to identify mKCs in the developing pupal worker brain. Areas corresponding to lKCs and sKCs, that had already stopped proliferating, are colored light green and purple, respectively. (Lower panels) *In situ* hybridization with *mKast* sense probe (control experiments) corresponding to the above sections. Note that *mKast* signals indicated by red arrowheads are detectable only in the P7 and P8 stages in the middle panels.

Next, we evaluated when and how these newly identified mKCs differentiate in the honeybee MBs during metamorphosis. We performed single *in situ* hybridization analysis for *mKast* using developing pupal worker brain sections. Honeybee pupae develop from the P1 to the P9 stage during metamorphosis before emergence [1]. First, during the P1 to P3 stages, lKCs differentiate from the cluster of neuroblastema cells located at the inner core of the developing MB calyces by asymmetric cell division, and migrate to the inside edges of the calyces. During the P3 to P5 stages, the sKCs also differentiate from the cluster of neuroblastema cells by asymmetric cell division [44]. Preferential *mKast*-expression became detectable in the upper area sandwiched by the lKCs and sKCs at the P7 stage, and was strongly detected in the same area at the P8 stage (Fig. 9). These results clearly indicate that mKCs, which are characterized by the preferential *mKast*-expression, begin to develop after the lKCs and sKCs cease to differentiate during metamorphosis.

## Discussion

### Identification of *mKast* homologs in honeybee and Hymenopteran insect genomes

In the present study, we first identified *mKast* (GB18367) that is preferentially expressed in the ‘L-2’/‘L-b’ lKCs, which we renamed ‘mKCs’. Although mKast is predicted to contain both arrestin-like_N and _C domains (Fig. 1), mKast had no apparent sequence identities with the predicted honeybee arrestins. Rather, mKast belonged to a protein superfamily distinct from the arrestin family and comprises mammalian ARRDCs (Fig. 2) [48]. In mammals, arrestins are a family of four proteins that regulate the signaling and trafficking of hundreds of different G-protein-coupled receptors: arrestin1 (called visual arrestin in some species), arrestin2 (also known as β-arrestin1), arrestin3 (β-arrestin2) and arrestin 4 (cone arrestin) [50]. Recent studies showed that β-arrestins serve as multiprotein scaffolds to bring elements of specific signaling pathways into close proximity [51]. More recently, mammalian ARRDCs with predicted ‘arrestin-like’ structural domains but lacking sequence homology have been identified to function like β-arrestin in receptor regulation [48]. Thus, it might be that mKast also has some roles in receptor regulation in mKCs and OL neurons expressing *mKast*.

In contrast, there are genes more closely related to *mKast* in the genomes of some Hymenopteran insects, such as *Apis florea*, the bumblebee, the alfalfa leafcutter bee, and *Nasonia vitripennis*. Our notion that *mKast* expression characterizes mKCs is consistent with the fact that *mKast* orthologs are conserved only in some Hymenopteran insects, including the honeybee, bumblebee and parasite wasp, which are equipped with elaborate MBs [22]. It is thus plausible that *mKast* homologs have acquired unique roles in the central nervous system of these Hymenopteran insects.

### Identification of mKCs that are characterized by *mKast* expression

We have so far identified many genes expressed in a lKC or sKC-preferential manner in the MBs of the honeybee brain [26]–[36]. Among them, *jhdk* and *trp* are unique in that they are preferentially expressed in both the outer lKCs and sKCs, but not in the inner lKCs, which we tentatively termed ‘L-2’ and ‘L-b’ lKCs for *jhdk* – and *trp-*expression analysis, respectively [41], [42]. The nature of the ‘L-2’/‘L-b’ lKCs, which we renamed ‘mKCs’, however, has remained obscure, because until now there has been no gene that is preferentially expressed in these specific KCs.

The present study revealed the unique characteristics of mKCs compared with lKCs and sKCs. First, they are located between lKCs and sKCs, and have an intermediate somata size compared with lKCs (7–9 µm) and sKCs (5–7 µm) [8] (Fig. 6). More importantly, the gene expression profiles of the mKCs are almost complementary to those of lKCs and sKCs: mKCs do not preferentially express *CaMKII* or *Mblk-1*, both of which are expressed in an lKC-preferential manner (Fig. 4), and they also do not preferenitally express *jhdk* nor *trp*, both of which are preferentially expressed in ‘L-1’/‘L-a’ lKCs ( = ‘real’ lKCs) and sKCs (Fig. 5). Detailed reexamination of our previous data regarding the genes expressed in a MB-preferential manner in the honeybee brain, other than *CaMKII* and *Mblk-1*, also confirmed that all of the genes that we reported to be expressed preferentially in lKCs, i.e., *IP_3_R*, *IP_3_K*, *BR-C*, *E75*, *reticulocalbin* and *ryr*, are actually expressed preferentially in ‘L-1’/‘L-a’ lKCs ( = ‘real’ lKCs) (data not shown). *mKast* is thus the only known gene that is expressed preferentially in mKCs in the honeybee MB at present.

### Some mKCs and sKCs are active in foraging workers

Our findings strongly suggest that neither preferential expression of *CaMKII* nor *mKast* characterizes the MB structures in the honeybee brain. Rather, a ‘clustering’ of neurons with similar gene expression profiles as particular KC types characterizes the honeybee MB structures. What, then, is the role of mKCs?

Neural activity mapping using *kakusei* revealed that, in addition to sKCs, some mKCs are more active in the forager brains (Fig. 8), suggesting their roles in visual information processing during the foraging flights [23]–[25]. We speculate that the ratio of active mKCs might vary depending on the extent of the neural activity of mKCs in the forager brains. To test this hypothesis, it would be interesting to analyze the ratio of active mKCs in foragers that have experienced different flight parameters, such as near or far food sources, flights associated with high or low optic flows [25], [52] or sugar-rich or sugar-poor food sources.

To understand how each KC type functions in higher visual information processing, it will be important to determine the projection patterns of each OL neuron type that preferentially expresses *CaMKII*, *mKast*, or neither, for example, by using indirect immunofluorescent staining with antibodies against CaMKII and mKast, or by introducing and expressing reporter genes that express fluorescent protein under the *CaMKII* or *mKast* promoters by using electroporation in the future studies [53].

### Differentiation of mKCs begins after lKCs and sKCs proliferation ceases

We also observed that, in contrast to sKCs and lKCs that differentiate from neuroblasts according to the time after birth, *mKast* expression became detectable after the cell division of the MB neuroblasts ceased (Fig. 9). Considering that mKCs are sandwiched between lKCs and sKCs, it is likely that mKCs are derived from some lKCs or sKCs that have already ceased to proliferate. It is not clear at all at present, however, whether mKCs are already committed during the cell division of the MB neuroblasts and then *mKast* expression becomes to be detectable, or whether mKCs are not yet committed during the cell division of the MB neuroblasts and then some lKCs or sKCs begin to express *mKast* to differentiate into mKCs. In the latter case, it might be that *mKast* expression suppresses the expression of genes that are expressed in an lKC or sKC-preferential manner, which creates mKC gene expression profiles that are almost complementary to those of lKCs and sKCs.

Our phylogenetic tree analysis suggested that *mKast* represents a gene that is unique to Aculeata Hymenopteran insects. Concomitantly, in Aculeata Hymenopteran insects, visual information processed in the OLs projects directly to the MBs. Therefore, comparison of gene expression profiles of *CaMKII* and *mKast* in the brains of various Hymenopteran insect species will be very important for future studies. We expect that analysis of the projection and function of mKCs, and molecular functions of mKast in the honeybee as well as the evolution of mKCs and mKast will provide critical insights into the evolution of the Hymenopteran insect brains and behaviors.

## Supporting Information

Figure S1 **Quantitative RT-PCR analysis of the** ***Clone #3*** **expression level in the OLs and the other brain regions.** The amounts of the *Clone #3* transcript normalized with that of the *EF-1alpha* transcript are indicated. Student's *t*-test was used for statistical analysis (**, *p*<0.01). Data are shown as the means ± SEM.
(TIFF)

Figure S2 **Double fluorescent** ***in situ*** **hybridization of** ***mKast*** **and** ***CaMKII*** **.** The same double *in situ* hybridization result with *CaMKII* and *mKast* antisense probes shown in Fig. 4D and Fig. 6. Merged image of nuclear signals detected by DAPI (blue), *mKast* signals detected by HNPP/FastRed (magenta), and *CaMKII-*signals detected by fluorescein (green) of the MBs that contained both lateral and medial calyces is shown. The medial calyces are located more frontally than the lateral calyces in the honeybee MBs [49]. Note that the area preferentially expressing *mKast* almost occupied inside of the area preferentially expressing *CaMKII* in the lateral calyx, which corresponded to the far front edge of the lateral calyx, whereas it was sandwiched between the area preferentially expressing *CaMKII* and the sKCs in the middle part of the medial calyx.
(TIFF)

Figure S3 ***In situ*** **hybridization of** ***mKast*** **in the nurse bee and forager MBs.** Nurse bee MB sections (right panels) and forager MB sections (left panels) hybridized with antisense probes (upper panels) or sense probes (lower panels, control experiments). Red arrowheads indicate *mKast* expression. (Upper right panel) Schematic drawing of lKCs (green), sKCs (blue) and mKCs expressing *mKast* (magenta). Note the similarity in the expression pattern of *mKast* between nurse bee and forager MBs.
(TIFF)

Figure S4 **Quantitative RT-PCR analysis of the** ***mKast*** **expression level in the nurse bee and forager brains.** The amounts of the *mKast* transcript normalized with that of the *EF-1alpha* transcript are indicated. Relative expression levels of *mKast* in the brain regions that mainly contained the MBs did not differ significantly between the nurse bees and foragers.
(TIFF)
