# Correction: Novel Middle-Type Kenyon Cells in the Honeybee Brain Revealed by Area-Preferential Gene Expression Analysis

**DOI:** 10.1371/annotation/1fa31a02-1b58-4361-98eb-5c213e5d5336

**Published:** 2013-11-13

**Authors:** Kumi Kaneko, Tsubomi Ikeda, Mirai Nagai, Sayaka Hori, Chie Umatani, Hiroto Tadano, Atsushi Ugajin, Takayoshi Nakaoka, Rajib Kumar Paul, Tomoko Fujiyuki, Kenichi Shirai, Takekazu Kunieda, Hideaki Takeuchi, Takeo Kubo

The paragraph that begins "In the latter experiment," appears incorrectly in the Introduction section. This paragraph should appear in the Results section as the third paragraph under the heading "Comparison of the mKast-expression profile with those of CaMKII and Mblk-1, which are expressed in an lKC-preferential manner."

Please see the full paragraph below:

In the latter experiment, however, the area preferentially expressing mKast was observed to simply cover the sKCs (Figs. 4F, H), rather than surrounding the upper regions of the sKCs (Fig. 4B, D). The honeybee MB calyces are shaped like baskets whose long sides are rather parallel with the sagittal axis of the brain [49], and the area inside the calyces, where the sKC somata exist, are shaped like cones, whose bases are somewhat elongated along the sagittal axis. Therefore, if frontal brain sections cross the MB calyces at the most front or most backward edges, they sometimes include mainly lKCs [36]. Similarly, it is plausible that, if frontal sections cross the MB calyces in the middle part, mKCs surround the sKCs, as shown in Fig. 4B and 4D, whereas, if the sections cross the MB calyces at the front or backward edges, mKCs merge to simply cover the sKCs, as shown in Figs. 4F and 4H. Actually, in the other case, the area preferentially expressing mKast almost occupied inside of the area preferentially expressing CaMKII in the lateral calyx (Fig. S2), which corresponded to the far front edge of the lateral MB calyx, whereas it was sandwiched between the area preferentially expressing CaMKII and the sKCs in the middle part of the medial MB calyx (Fig. S2, 4B, D). 

