# Supplementary figures and images for: Novel Middle-Type Kenyon Cells in the Honeybee Brain Revealed by Area-Preferential Gene Expression Analysis
Source: PLoS One. 2013 Aug 21;8(8):e71732. doi: 10.1371/journal.pone.0071732 (PMC3749211; doi:10.1371/journal.pone.0071732)

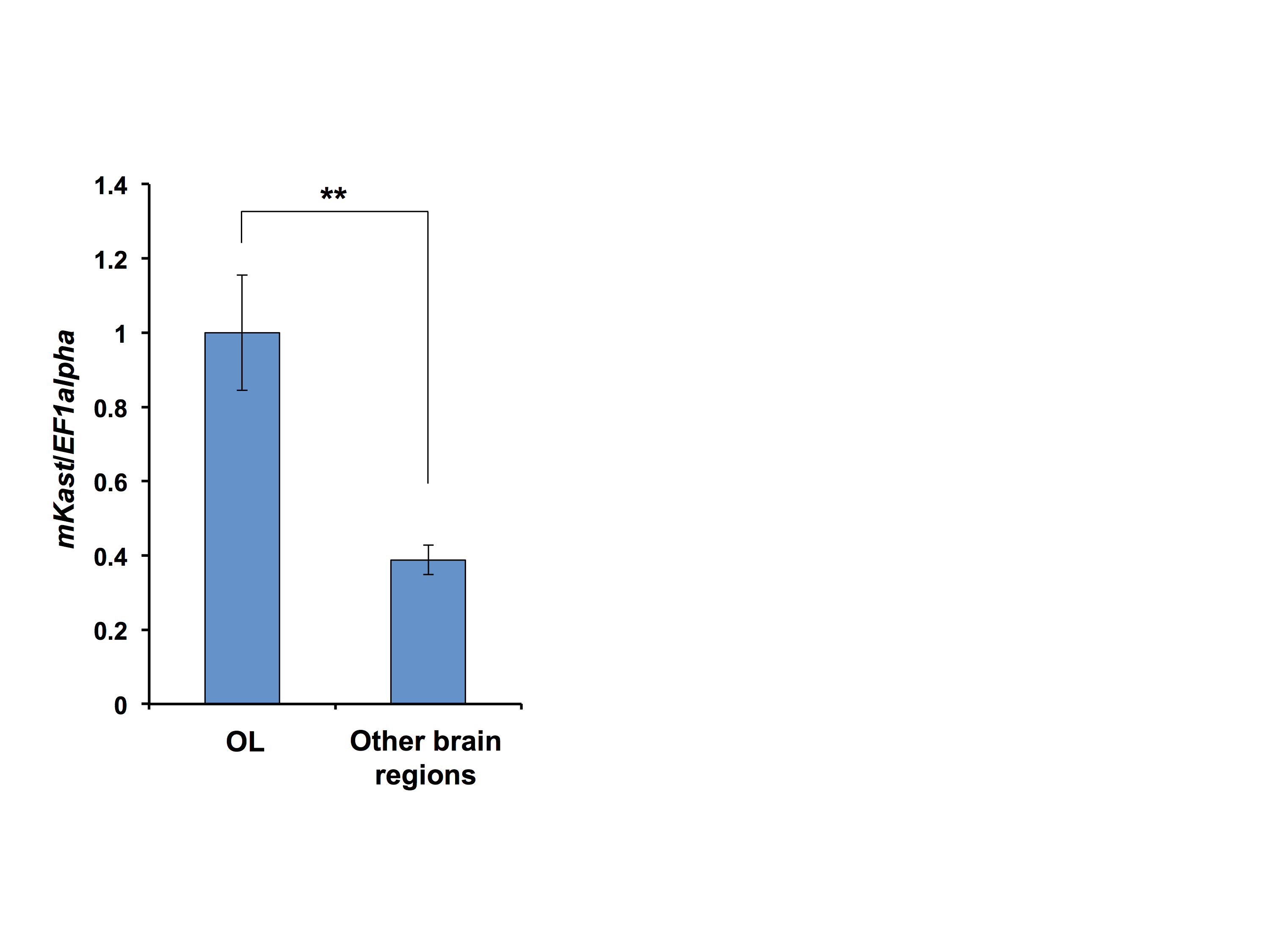

Supplement: Figure S1 — Quantitative RT-PCR analysis of the Clone #3 expression level in the OLs and the other brain regions. The amounts of the Clone #3 transcript normalized with that of the EF-1alpha transcript are indicated. Student's t-test was used for statistical analysis (**, p<0.01). Data are shown as the means ± SEM. (TIFF) [file pone.0071732.s001.tiff]

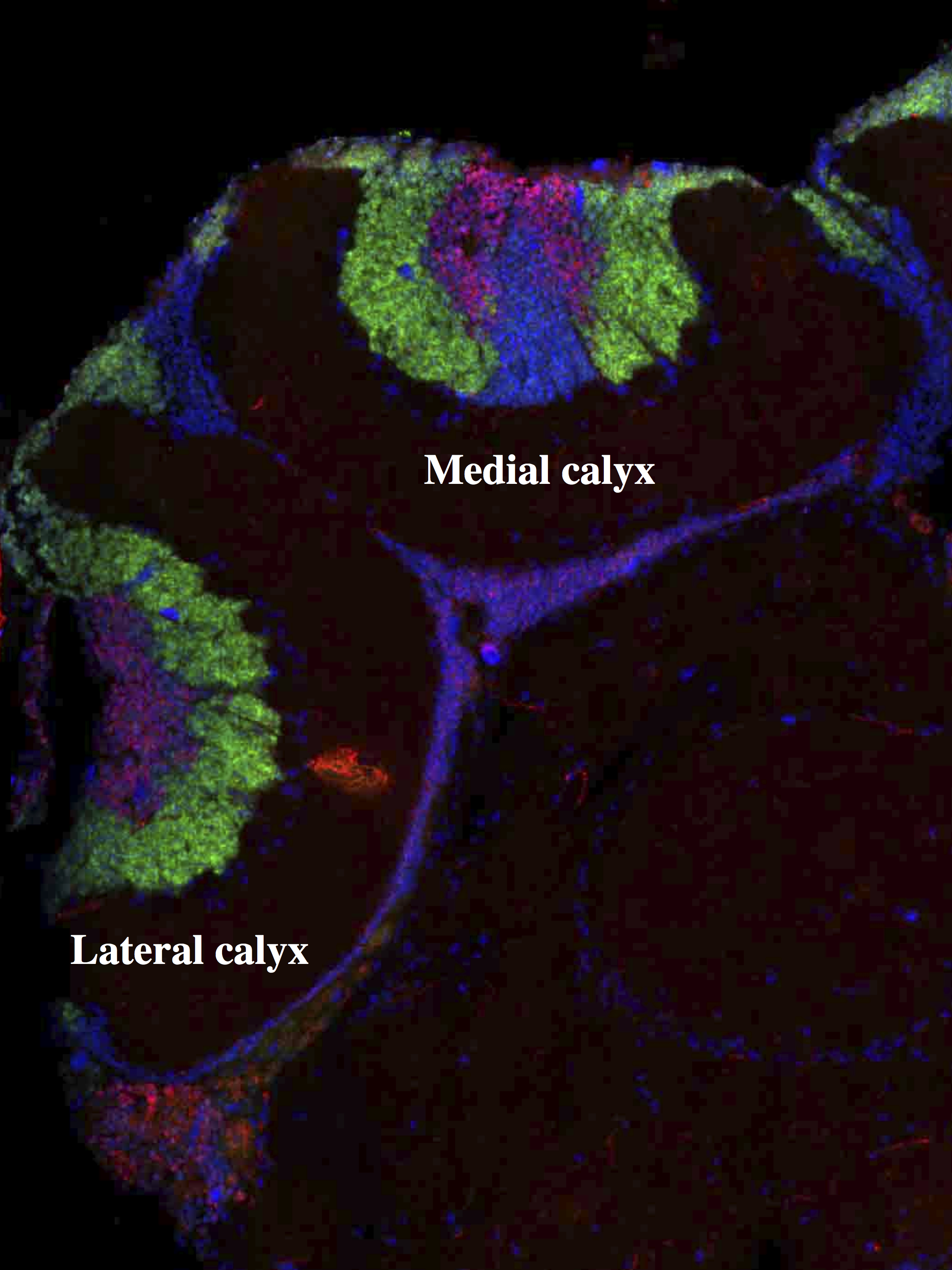

Supplement: Figure S2 — Double fluorescent in situ hybridization of mKast and CaMKII . The same double in situ hybridization result with CaMKII and mKast antisense probes shown in Fig. 4D and Fig. 6. Merged image of nuclear signals detected by DAPI (blue), mKast signals detected by HNPP/FastRed (magenta), and CaMKII-signals detected by fluorescein (green) of the MBs that contained both lateral and medial calyces is shown. The medial calyces are located more frontally than the lateral calyces in the honeybee MBs [49]. Note that the area preferentially expressing mKast almost occupied inside of the area preferentially expressing CaMKII in the lateral calyx, which corresponded to the far front edge of the lateral calyx, whereas it was sandwiched between the area preferentially expressing CaMKII and the sKCs in the middle part of the medial calyx. (TIFF) [file pone.0071732.s002.tiff]

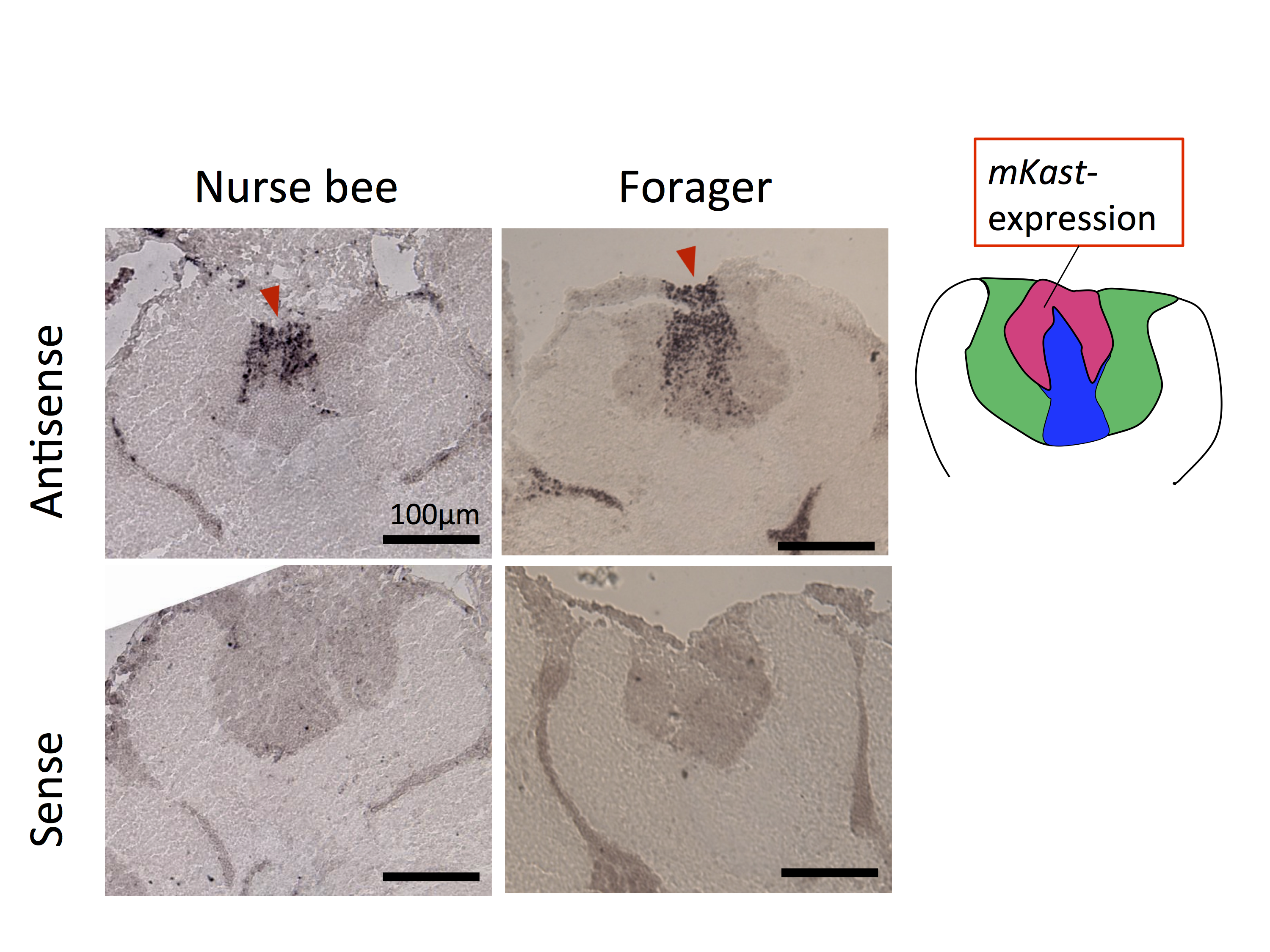

Supplement: Figure S3 — In situ hybridization of mKast in the nurse bee and forager MBs. Nurse bee MB sections (right panels) and forager MB sections (left panels) hybridized with antisense probes (upper panels) or sense probes (lower panels, control experiments). Red arrowheads indicate mKast expression. (Upper right panel) Schematic drawing of lKCs (green), sKCs (blue) and mKCs expressing mKast (magenta). Note the similarity in the expression pattern of mKast between nurse bee and forager MBs. (TIFF) [file pone.0071732.s003.tiff]

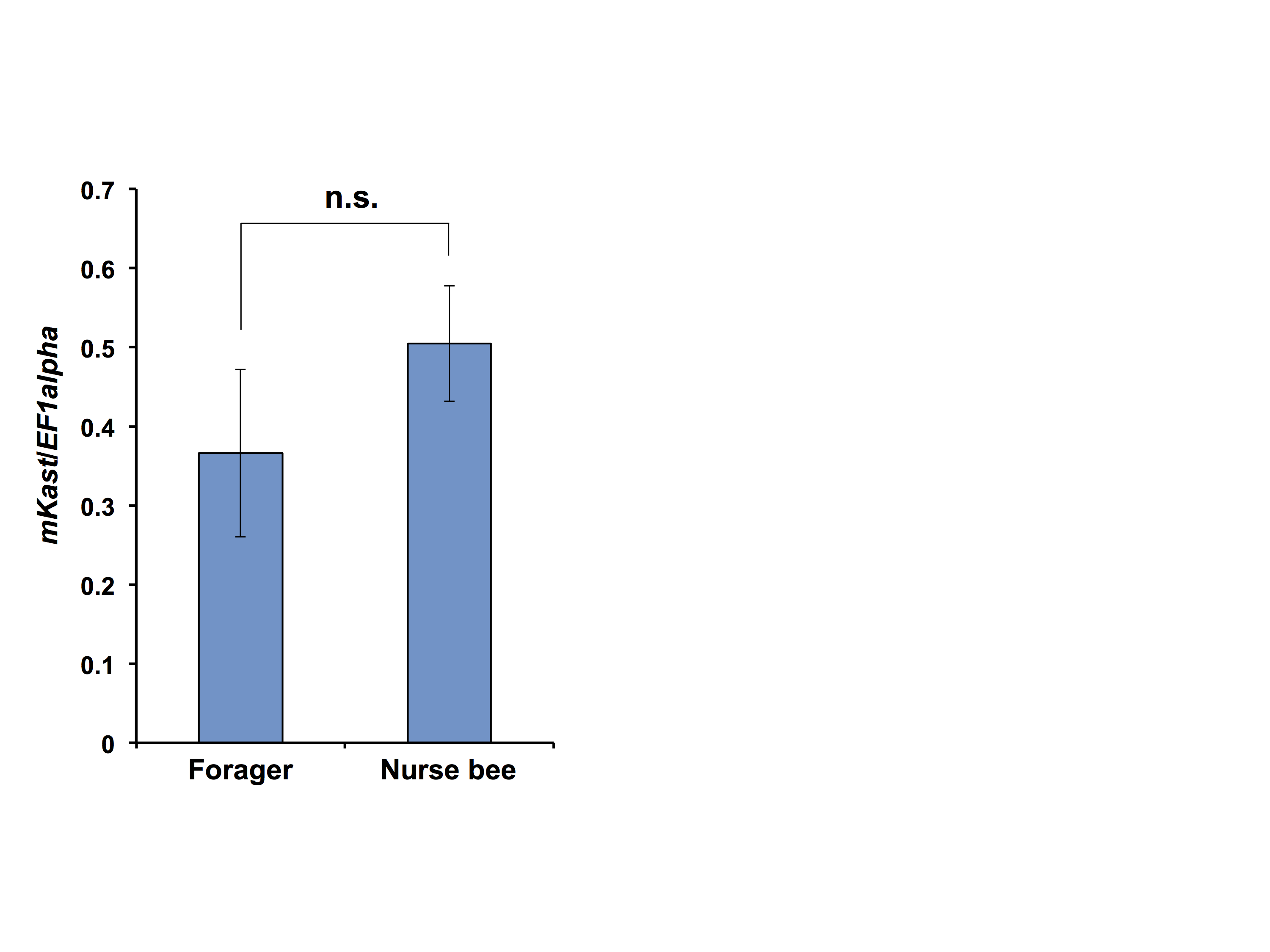

Supplement: Figure S4 — Quantitative RT-PCR analysis of the mKast expression level in the nurse bee and forager brains. The amounts of the mKast transcript normalized with that of the EF-1alpha transcript are indicated. Relative expression levels of mKast in the brain regions that mainly contained the MBs did not differ significantly between the nurse bees and foragers. (TIFF) [file pone.0071732.s004.tiff]
